# Subcortical pathways to extrastriate visual cortex underlie residual vision following bilateral damage to V1

**DOI:** 10.1016/j.neuropsychologia.2018.01.007

**Published:** 2019-05

**Authors:** Sara Ajina, Holly Bridge

**Affiliations:** Wellcome Centre for Integrative Neuroimaging, FMRIB, Nuffield Department of Clinical Neurosciences, University of Oxford, UK

**Keywords:** Hemianopia, Cortical blindness, V1, Visual perception

## Abstract

Residual vision, or blindsight, following damage to the primary visual cortex (V1) has been investigated for almost half a century. While there have been many studies of patients with unilateral damage to V1, far fewer have examined bilateral damage, mainly due to the rarity of such patients. Here we re-examine the residual visual function and underlying pathways of previously studied patient SBR who, as a young adult, suffered bilateral damage restricted to V1 which rendered him cortically blind. While earlier work compared his visual cortex to healthy, sighted participants, here we consider how his visual responses and connections compare to patients with unilateral damage to V1 in addition to sighted participants.

Detection of drifting Gabor patches of different contrasts (1%, 5%, 10%, 50% and 100%) was tested in SBR and a group of eight patients with unilateral damage to V1. Performance was compared to the neural activation in motion area hMT+ measured using functional magnetic resonance imaging. Diffusion tractography was also used to determine the white matter microstructure of the visual pathways in all participants.

Like the patients with unilateral damage, patient SBR showed increased % BOLD signal change to the high contrast stimuli that he could detect compared to the lower contrast stimuli that were not detectable. Diffusion tractography suggests this information is conveyed by a direct pathway between the lateral geniculate nucleus (LGN) and hMT+ since this pathway had microstructure that was comparable to the healthy control group. In contrast, the pathway between LGN and V1 had reduced integrity compared to controls. A further finding of note was that, unlike control participants, SBR showed similar patterns of contralateral and ipsilateral activity in hMT+, in addition to healthy white matter microstructure in the tract connecting hMT+ between the two hemispheres. This raises the possibility of increased connectivity between the two hemispheres in the absence of V1 input.

In conclusion, the pattern of visual function and anatomy in bilateral cortical damage is comparable to that seen in a group of patients with unilateral damage. Thus, while the intact hemisphere may play a role in residual vision in patients with unilateral damage, its influence is not evident with the methodology employed here.

## Highlights

• Bilaterally hemianopic patient SBR has neural patterns like unilateral patients.
• hMT+ activity increases with stimulus contrast and better stimulus detection.
• Like in unilateral patients, the pathway between LGN and hMT+ is intact in SBR.

## 1 Introduction

For almost half a century there has been debate around how patients with damage to the primary visual cortex (V1), the main input to the visual brain, maintain some ability to see, that may be conscious or unconscious (Cowey, 2010, Weiskrantz et al., 1974). The pathways hypothesised to underlie these blindsight abilities, however appear to be weak in the healthy visual system (Schmid et al., 2010, Sincich et al., 2004, Warner et al., 2015), and therefore make a difficult target for investigation. The advent of functional magnetic resonance imaging (fMRI) has strengthened the evidence supporting the human motion area hMT+ as a likely visual structure to underlie this residual function (Barbur et al., 1993, Goebel et al., 2001, Morland et al., 2004, Zeki and Ffytche, 1998).

The early studies using fMRI identified hMT+ as a region that was activated by moving stimuli even when presented within the cortically blind region (Barbur et al., 1993, Goebel et al., 2001, Morland et al., 2004). Recent work on patients with unilateral damage to V1 has indicated, however, that although hMT+ is activated in many patients the pattern of response is abnormal and resembles that seen in V1 of sighted people (rather than hMT+) (Ajina et al., 2015a, Ajina et al., 2015c). This suggests that the input may be comparable to V1, and therefore the response reflects direct subcortical input. To support this hypothesis, diffusion tractography has shown that the pathway between the lateral geniculate nucleus (LGN) and hMT+ in patients with unilateral hemianopia is comparable to healthy control subjects only in patients who show blindsight. In hemianopic patients without blindsight the pathway between LGN and hMT+ shows a deficit in white matter microstructure (Ajina et al., 2015b). Thus, there appears to be a link between the integrity of this specific pathway and the presence of blindsight. In contrast, both the pathway between hMT+ in the two hemispheres, and the pathway between the superior colliculus and hMT+ did not differ between those patients with and without blindsight.

In patients with unilateral damage to V1, the intact hemisphere may also play a role in the residual function, or blindsight. An example of this phenomenon was seen in Patient GY who perceived moving phosphenes in his blind hemifield only when transcranial magnetic stimulation (TMS) was applied to his intact hemisphere in addition to his lesioned one (Silvanto et al., 2007). This suggests that a contribution from the intact hemisphere is required to boost perception. In contrast, some studies have indicated that the intact hemisphere has abnormal responses to visual stimulation, term ‘sightblind’ (Bola et al., 2013). Hess and Pointer (1989), for example, demonstrated a decrease in contrast sensitivity in the intact hemisphere of hemianopic patients.

In an extreme case where the right hemisphere did not develop in a child, the occipital cortex of the left hemisphere developed maps of both the contralateral right hemifield *and* the ipsilateral left hemifield (Muckli et al., 2009). A similar phenomenon was found in two later studies of hemianopic children (Mikellidou et al., 2017, Tinelli et al., 2013) indicating that, where plasticity is maximised, the intact hemisphere is able to adapt its function to compensate for cortical visual damage.

Patients with bilateral damage to V1 are fortunately rare, but can provide an opportunity to understand whether pathways in the damaged hemisphere are sufficient to underlie residual vision and blindsight or whether the intact side is required. Patient TN, described as having ‘affective blindness’ has extensive damage to the occipital lobe bilaterally, due to sequential strokes, but can nonetheless navigate (de Gelder et al., 2008), detect human bodies (Van den Stock et al., 2014) and looming motion (Hervais-Adelman et al., 2015). A second patient with bilateral V1 damage is SBR whose lesions are restricted to V1 leaving extrastriate visual cortex intact (Bridge et al., 2010). This patient shows evidence of residual function in his central visual field, requiring high contrast (>50%) for detection, consistent with unilateral patients with blindsight (Ajina et al., 2015c). This suggests that his performance may be comparable to patients with unilateral damage. A further parallel with unilateral damage is indicated by a recent study of a child with bilateral damage to the occipital cortex who shows remarkably intact visual behaviour (Mundinano et al., 2017).

The current study aims to determine the pattern of response to visual stimulation in hMT+ of patient SBR compared to both patients with unilateral damage and healthy control participants. Specifically, we quantified the blood-oxygenation-level-dependent (BOLD-) signal change in hMT+ to stimuli of high and low contrast in both the ipsi- and contra-lateral hemispheres. Furthermore, using diffusion tractography we demonstrated that the microstructure of both the tract between LGN and hMT+ and between hMT+ in the two hemispheres is comparable to healthy control participants, suggesting that both these tracts may be important when damage is bilateral.

## 2 Materials and methods

### 2.1 Participants

**Fig. 1:**
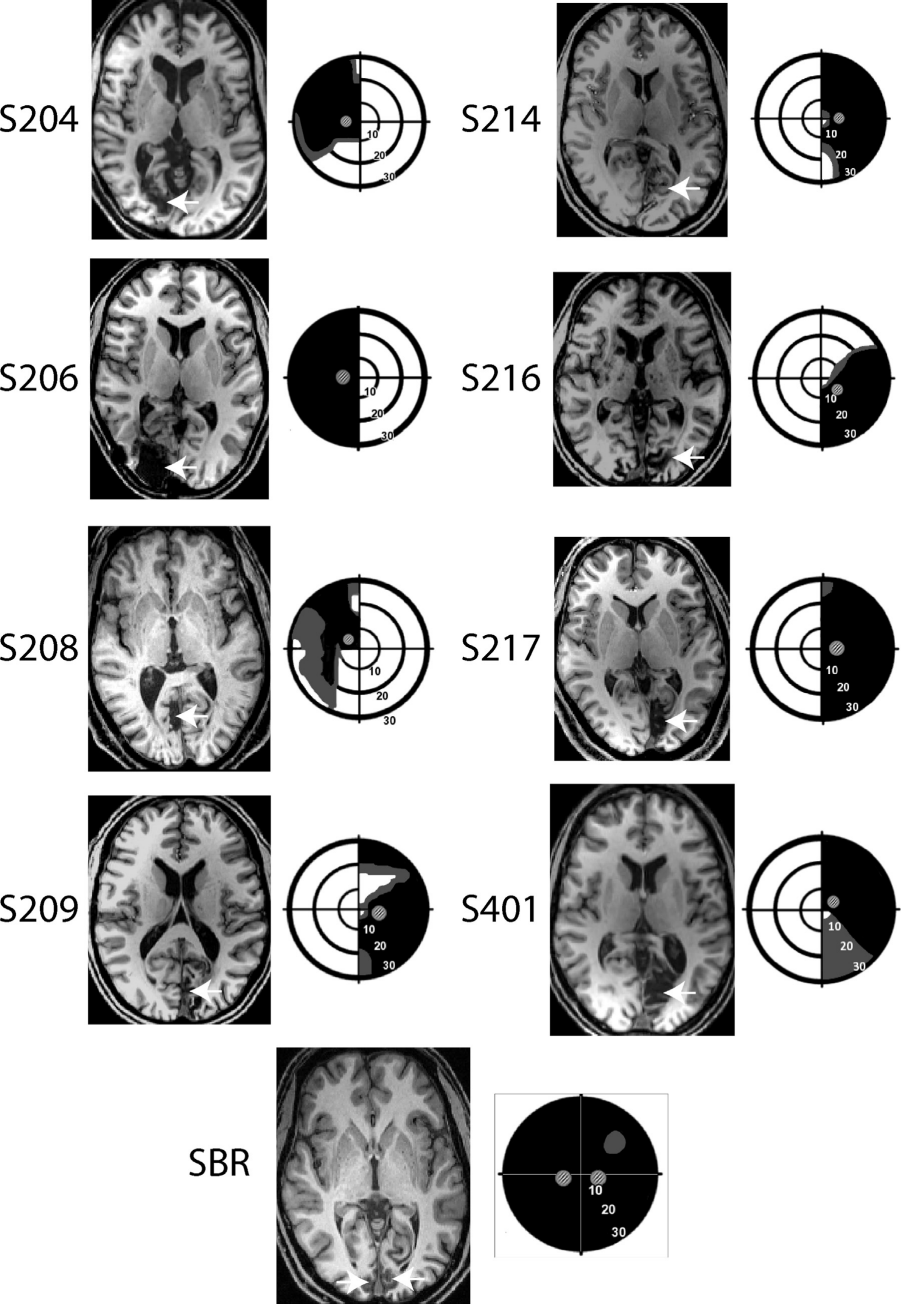
The lesion and visual field loss is shown for each patient with unilateral hemianopia and SBR. The arrows indicate the location of the lesion for each patient. The visual fields are presented schematically based upon Humphrey perimetry, with black indicating most severe visual loss and grey indicating partial loss. The visual fields of S216 are based on Goldmann perimetry. The small Gabor patches indicate the location in which the visual stimuli were presented to the patient.

Eight patients (three females) with unilateral damage to V1 were recruited to the study. Each had a significant reduction of the visual fields, with a minimum quadrant loss of vision, as quantified with Humphrey perimetry. Average age was 50 years ± 15.4 sd. and all infarcts had been incurred at least 6 months prior to recruitment to the study. Structural images indicating the location of the lesion in each patient are shown in Fig. 1.

Patient SBR suffered a hypoxic event of unknown origin at the age of 22 which left him clinically blind. He has been studied previously and was 29 at the time of scanning. His bilateral lesions to the primary visual cortex are shown in Fig. 1, along with his visual fields measured with Humphrey perimetry. The lesion is substantially less extensive than the lesions in the patients with unilateral damage, despite the large field loss.

In addition, 8 participants with healthy vision were recruited to act as controls. Average age for these control participants was 53.2 years ± 12.1 sd.

All participants provided written consent and ethical approval was provided by the Oxfordshire Research Ethics Committee (Ref B08/H605/156).

### 2.2 Visual stimulation

For both behavioural and MRI experiments, the visual stimulus was a drifting achromatic Gabor patch of either 5° or 8° (depending on the area of visual loss) displayed on a uniform grey background of luminance 50 cd/m^−2^ (equivalent to the mean luminance of the Gabor patch). The spatial frequency was 1.3 cycles/° and temporal frequency was 10 Hz, with five contrast levels used (1%, 5%, 10%, 50% and 100%). The stimulus position was matched to the location of the scotoma in each patient individually and the corresponding position in the sighted hemifield. The location of the stimuli related to the scotoma is shown in Fig. 1. Psychophysical testing of visual performance was undertaken only in the patients, as those with healthy vision would perform at ceiling. For the fMRI testing in the healthy control group, stimulus size and position were matched to the patient group in a pairwise manner.

### 2.3 Behavioural testing procedure

**Fig. 2:**
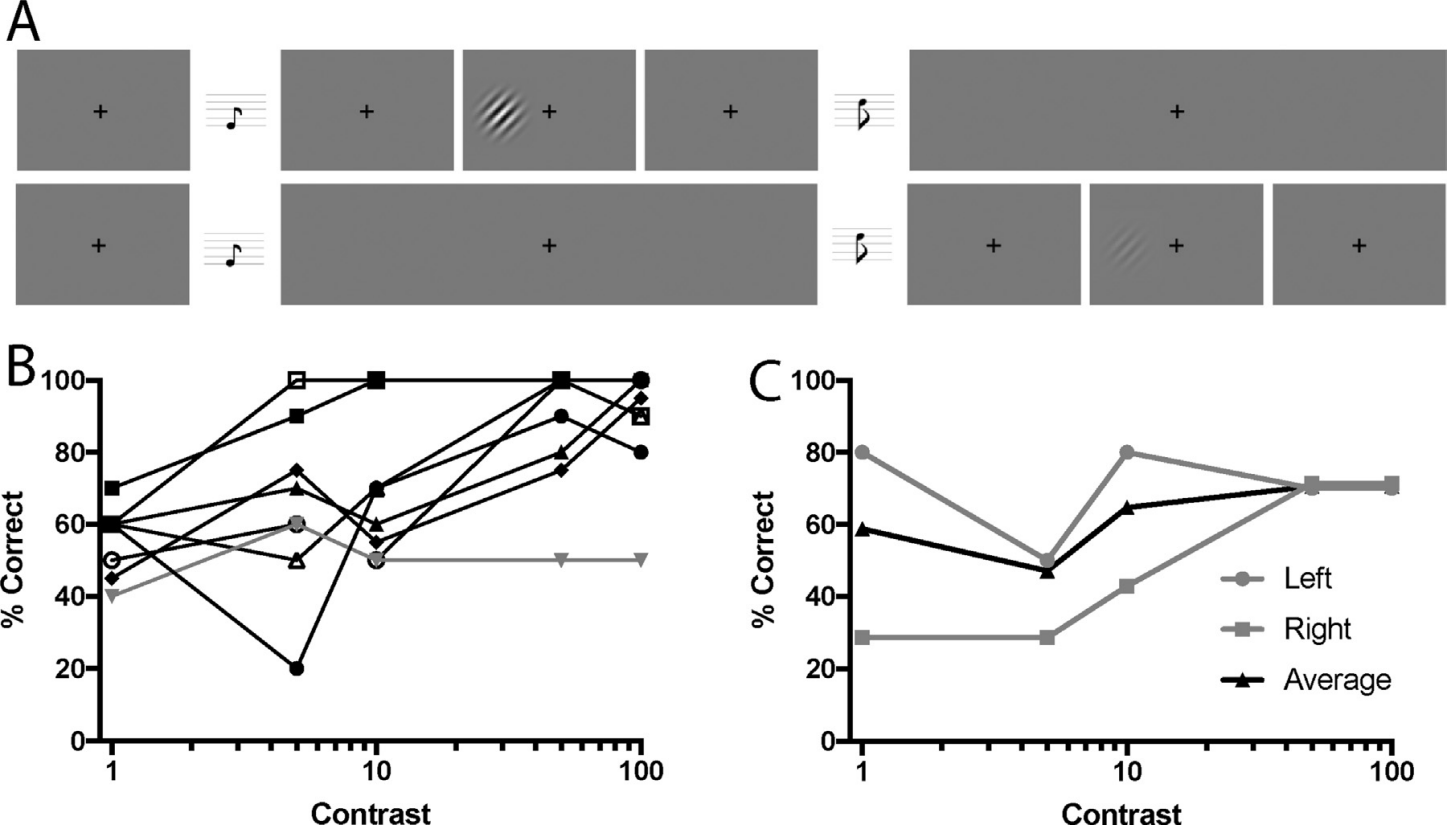
A shows the psychophysical task performed by the patients with unilateral homonymous hemianopia (n = 8) and patient SBR who has bilateral cortical blindness. The patients were required to determine which of the two intervals contained the Gabor patch. The upper row shows a high contrast Gabor patch, while the lower row shows a low contrast Gabor patch. In each case the Gabor patch is only presented for 500 ms rather than the entire trial. B shows the performance of each of the unilateral patients at the different contrast levels. Only one patient (grey line) was unable to perform this task above chance when the stimulus was at 100% contrast. C shows the performance of SBR in the two hemifields, and indicates his ability to perform above chance at the higher two contrasts.

Psychophysical testing was conducted outside the MRI scanner, with a 60 Hz CRT monitor at a distance of 68 cm. Participants performed a 2-alternative forced choice (2-AFC) task, indicating whether the Gabor patch was present in the first or second interval (Fig. 2A). They were instructed to guess if they did not see anything in either interval. Auditory tones of 300 Hz marked the onset of the first interval and 1200 Hz for the second. The Gabor patch appeared for 500 ms with jittered onset while the participant fixated a central black cross. The five different stimulus contrasts were presented at random, with 20 trials per level. Participants also performed a run of control testing in which stimuli were presented to the equivalent location in the sighted visual field. Fixation was monitored through the testing with an Eyelink 1000 eye tracker, and any trials in which eye position exceeded 1° from fixation were excluded from analysis. SBR was unable to see the fixation cross, but was able to maintain central gaze using verbal cues from the examiner, who monitored eye gaze in real time with the eye tracker.

### 2.4 fMRI visual stimulation procedure

Participants viewed a 1280 × 1040 resolution monitor at the back of the MRI scanner bore via a double mirror mounted on the head coil. The same five contrast levels were used, but rather than being randomised, each contrast was presented for a block of trials. This meant that in each block, a Gabor of a given contrast and hemifield appeared eight times with a duration of 2 s and an interstimulus interval of 500 ms. Since there were 5 contrast levels and each appeared on both left and right hemifield, there were 10 distinct blocks. In each case, the stimulus was the same size and presented in the same location as for the behavioural testing. A 10 s rest period with a mid-grey background followed each 20 s block. In total, participants performed 3 runs of the experiment, each of which lasted 300 s.

To ensure participants did not look at the stimuli, they were required to monitor the fixation cross and press a button when the colour changed from black to red. Colour changes lasted 300 ms and occurred at random. Participants were told not to miss any red crosses and that they must try to maintain fixation throughout the experiment, not moving their eyes around the screen. For details of performance in unilateral patients and control participants, see Ajina et al. (2015c). An EyeLink 1000 eye tracker was used to confirm central fixation.

### 2.5 MRI acquisition and preprocessing

Scanning took place in a 3T Siemens Verio MRI scanner at the Functional MRI of the Brain (FMRIB) Centre (University of Oxford), using a 32 channel head coil.

#### 2.5.1 Anatomical scanning

A structural scan was acquired for each participant for registration purposes and to quantify the location and size of the lesion. This was a high-resolution (1 mm isotropic) whole head T1-weighted MPRAGE anatomical image (TE=4.68 ms; TR=2040 ms; field of view=100 mm; flip angle=8°).

#### 2.5.2 Functional MRI

Four hundred fifty-six functional volumes were acquired in a single session, duration 15 min [T2*-weighted EPI; 34 sequential 3 mm slices; repetition time (TR), 2000 ms; echo time (TE), 30 ms; field of view, 192 mm]. Magnetisation was allowed to reach a steady state by discarding the first five volumes, an automated feature of the scanner. A field map with dual TE images (TE1, 5.19 ms; TE2, 7.65 ms; whole-brain coverage; voxel size, 2 × 2 × 2 mm^3^) was also acquired for each participant to reduce spatial distortion. Preprocessing and statistical analyses were performed using tools from FSL (FMRIB Software Library, http://www.fmrib.ox.ac.uk/fsl). Non-brain tissue was excluded from analysis using BET (Brain Extraction Tool; (Smith, 2002)), motion correction was performed using MCFLIRT (FMRIB Linear Image Restoration Tool with Motion Correction; (Jenkinson et al., 2002, Jenkinson and Smith, 2001), images were corrected for distortion using field maps, spatial smoothing used a Gaussian kernel of FWHM of 5 mm, and highpass temporal filtering (Gaussian-weighted least-squares straight-line fitting, with 13.0 s) was used. Functional images were registered to high-resolution structural scans using FLIRT (FMRIB Linear Image Restoration Tool; Jenkinson and Smith, 2001) and to a standard MNI brain template using FLIRT and FNIRT (FMRIB Nonlinear Image Registration Tool; Andersson et al., 2007).

### 2.6 Diffusion-weighted MRI

EPI images at a resolution of 2 × 2 × 2 mm^3^ (TR=8900 ms; TE=91.2 ms) were acquired with a diffusion weighting isotropically distributed along 60 directions (b-value = 1500 s/mm^2^). Four non-DWI (B0) images were acquired in total, one every 16 volumes. To minimise the effects of geometric distortions, two image sets were acquired with the phase-encoded direction reversed, ‘blip-up’ and ‘blip-down’ (Chang and Fitzpatrick, 1992). The image sets have geometric distortions of equal size but opposite direction, which allows for the calculation of a corrected image (Andersson and Sotiropoulos, 2016). Before correcting for geometric distortions, each image set, blip-up and blip-down, was corrected for motion and eddy-current related distortions, using tools from FSL (http://www.fmrib.ox.ac.uk/fsl). The corrected images were concatenated in time and aligned to the motion-corrected mean of the B0 images using a rigid body algorithm. Dw-MRI images were then aligned to the T1 structural scan.

### 2.7 Data analysis

#### 2.7.1 Regions of interest

Masks for hMT+ were derived from anatomically defined probabilistic maps (Juelich atlas implemented in FSL; (Allen et al., 2015; Pestilli et al., 2014; Yeatman et al., 2012)), nonlinearly transformed into functional and diffusion space for all participants. V1 masks in controls and in the intact hemisphere of patients with unilateral damage were defined functionally for each participant so that they corresponded to stimulated regions of calcarine cortex. An equivalent region was drawn in the damaged hemisphere. In SBR, where this was not possible, V1 was defined anatomically and corresponded to a comparable volume to unilateral patients and controls. LGN masks were defined anatomically by manual inspection of each structural image individually. The average mask size was 246 mm^3^ and 237 mm^3^ for the left and right LGN of the control group, 248 mm^3^ for the intact hemisphere of the unilateral patients, 248 mm^3^ for the lesioned hemisphere and 266 mm^3^ and 202 mm^3^ for left and right hemispheres of SBR.

#### 2.7.2 fMRI analyses

Each of the 10 fMRI blocks described above (i.e., left and right hemifield at 5 contrast levels) were entered into the general linear model (GLM) as separate explanatory variables and were contrasted against the baseline fixation task to generate contrast of parameter estimates for each condition in every voxel. Parameter estimates modelling signal change were then extracted from regions of interest (ROIs) within functional space for each individual. The percentage signal change was calculated by scaling each contrast by the peak–peak height of the regressor and dividing by the mean over time. These measures were averaged across participants to generate group plots for signal change as a function of stimulus contrast. 1%, 5% and 10% contrasts were then averaged to form a ‘low contrast’ stimulus and 50% and 100% to give a ‘high contrast’ value.

#### 2.7.3 Diffusion data analysis

##### 2.7.3.1 Fascicle tracking

The details of tracking have been described elsewhere (Ajina et al., 2015b), but are described in brief here. The tracking algorithm was restricted to the white matter, which was manually edited where necessary. To account for crossing fibres (Frank, 2001, Frank, 2002, Rokem et al., 2015), so-called fibre orientation distribution functions (fODF) were estimated in each white matter voxel using constrained spherical deconvolution (CSD; (Tournier et al., 2007)). A response function, representing the signal of a single coherent bundle of nerve fibres, was estimated as a lower-order (Lmax = 4) CSD fit to the signal from voxels in which FA was larger than 0.7. CSD was then fit to the entire white matter with this response function and maximum harmonic order (Lmax) was set to 8 (see Ajina et al. (2015b) for full explanation). A probabilistic ‘region to region’ algorithm implemented in MRtrix (Tournier et al., 2007) was used for fascicle tracking on the fODFs estimated with CSD (Tournier et al., 2007). Streamlines were generated from 10,000 seeds inside a union mask created by the combination of two ROIs. Streamlines had to touch both ROIs and travel only within white matter to be included in the output. A curvature radius threshold of 1 mm and step size of 0.2 mm was used. The total number of streamlines generated was constrained to a maximum of 1000,000. If there was a failure to adequately track any streamlines between hMT+ bilaterally (as occurred in 5 patients and 4 controls), a union mask was created between either left or right hMT+ and a cross-section of the corpus callosum. Streamlines were then tracked separately for each hemisphere, and recombined to represent the entire hMT+ <-> hMT+ pathway.

##### 2.7.3.2 Anatomically-informed identification of the tracts of interest

After fascicles were reconstructed for each pathway of interest, we used an anatomically informed approach to identify core-fascicles to compare across individuals (Ajina et al., 2015b, Allen et al., 2015, Pestilli et al., 2014, Yeatman et al., 2012). Outlier steamlines were removed from tracts in each brain to retain a ‘cleaned’ core bundle representing the most conservative estimate of the tract. To identify outlier streamlines, we calculated the Mahalanobis distance of nodes in each streamline from the core fascicle bundle, and removed streamlines located more than 2.6 standard deviations away from the core of the tract, and more than 2.8 standard deviations longer than the mean tract length, using a Gaussian distribution to represent fascicle distance and length.

##### 2.7.3.3 Tract-based microstructure and statistical testing

The diffusion tensor model (Basser et al., 1994) was used to derive fractional anisotropy (FA) maps, which were used as a measure of ‘white matter microstructure’ (as described in Ajina et al. (2015b)). FA provides a measure of the directionality of water molecule movement, which relates to the geometric organization of axons and fascicles in each voxel (e.g., crossing, merging or ‘kissing’ fibres), the degree of myelination of axons in the white matter (Beaulieu and Allen, 1994), and their packing density (Sen and Basser, 2005). In cases of brain damage, a decrease in FA can be indicative of loss of structural integrity of fibre bundles (Jones et al., 2013) such as Wallerian degeneration (Beaulieu and Allen, 1994).

Measures of tract integrity were carried out using ‘cleaned’ fascicle bundles, and tracts were processed using software routines part of MBA (Matlab Brain Anatomy: https://github.com/francopestilli/mba). We selected three tracts of interest, between: (1) LGN and V1, and (2) LGN and hMT+ in the same hemisphere, and (3) between hMT+ bilaterally.

In order to compare values across participants, a standardised measure was derived for each tract. The voxel-wise tensor FA was combined with the spatial information of the trajectory of tracts within the white matter to compute a tract profile (see Ajina, Pestilli, et al., Materials and Methods for full details). As performed previously, tracts were resampled to 100 nodes and the regions between nodes 15–85 were used to represent ‘whole tract’ profiles, and 85–100 for the distal region, while ensuring that the measures were not contaminated by grey matter voxels.

## 3 Results

To quantify visual performance in patients with V1 damage, the ability to detect a Gabor patch of different contrasts was determined using a 2-AFC task (Fig. 2A). Participants were considered to exhibit blindsight if they could detect the Gabor patches significantly above chance either across all five contrast levels, or for the 100% contrast condition. Using these criteria, seven of the eight patients with unilateral hemianopia were considered to have blindsight (Fig. 2B), with the patient without blindsight shown in grey. Patient SBR (Fig. 2C) was also able to detect the stimulus above chance in both hemifields at 50% and 100% contrast.

### 3.1 BOLD response is significantly greater for high contrast stimuli that were detectable in SBR and patients with unilateral damage

**Fig. 3:**
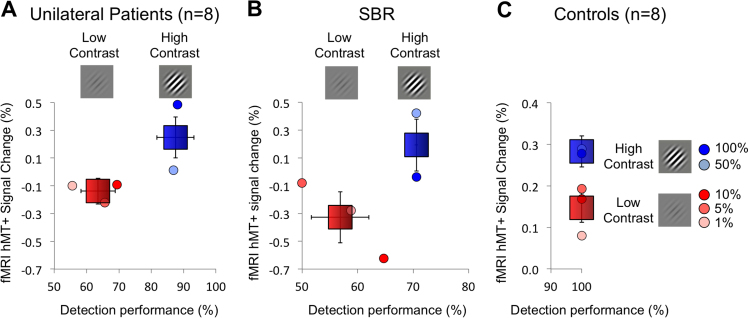
In the blind hemifield of the 8 patients with unilateral damage to V1 and in patient SBR with bilateral V1 damage, the %BOLD change in hMT+ to high contrast stimuli (50% and 100%) is significantly higher than that to low contrasts (1%, 5%, 10%). Healthy control participants (n=8) also show a higher response to the high contrast stimuli, but performance would be at ceiling for both high and low contrast detection (note the different scale on both axes). Signal change and performance scores are also provided for each contrast level separately.

To determine whether there was any link between detection of the stimulus and cortical activity to contrast level, the % BOLD signal change in hMT+ was calculated for ‘low’ contrast (1%, 5% and 10%) and ‘high’ contrast (50% and 100%). Fig. 3A shows the relationship between psychophysical detection and percent signal change in the blind hemifield of unilateral hemianopia patients. These patients showed significantly greater signal in the high contrast conditions compared to the low contrast (t = 2.4; d.f. = 38; p = 0.02). Similarly, in SBR the response across the two hemifields was significantly greater at high contrast than at low contrast (t = 3.7; d.f. = 8; p = 0.006). Fig. 3C shows the percent signal change to the low and high contrast stimuli in the healthy control participants. Psychophysical data were not acquired for this group as all would have performed at ceiling. Interestingly, the level of activity to the high contrast stimuli is similar across patients and controls. In comparison, the signal change to the low contrast stimuli is negative in the patients but significantly above zero in the controls. There appears to be a strong relationship between the neural activity to stimulus contrast in hMT+ and behavioural performance in both unilateral hemianopia and bilateral patient SBR.

### 3.2 SBR shows intact tracts between LGN and hMT+ and between hMT+ in the two hemispheres

**Fig. 4:**
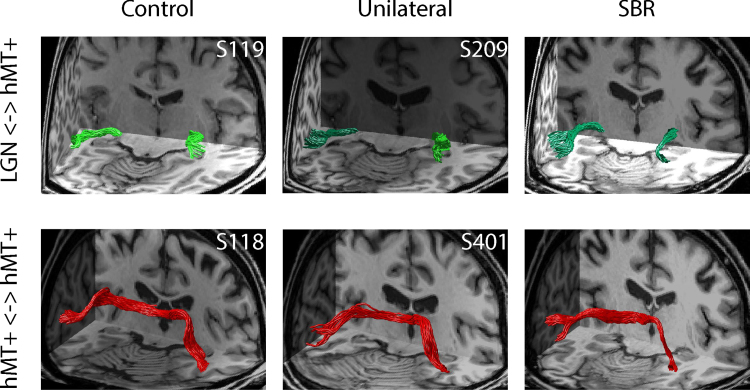
Tracts between LGN and hMT+ were identified in control participants, patients with unilateral hemianopia and SBR (upper row). Tracts in healthy hemisphere are shown in light green, whereas those in the damaged hemisphere are dark green. Tracts between hMT+ in the two hemispheres were also determined (lower row), shown in red. (For interpretation of the references to color in this figure legend, the reader is referred to the web version of this article.)

To investigate whether the increased signal change to visual stimulation was related to the underlying anatomy, diffusion imaging was used to investigate the microstructure of tracts projecting to area hMT+. Specifically, in a comparable way to our previous paper, we ran tracts between LGN and hMT+ and between hMT+ in the two hemispheres. Fig. 4, upper row, shows the tracts between LGN and hMT+ in an example control participant, a patient with unilateral damage to V1 (dark green tract) and SBR. The lower row shows the tracts running between hMT+ in the two hemispheres, which may be used to communicate information presented to the two sides of the visual field.

### 3.3 The distal parts of the LGN <-> hMT+ tract are less affected than regions near to LGN in SBR and unilateral patients

**Fig. 5:**
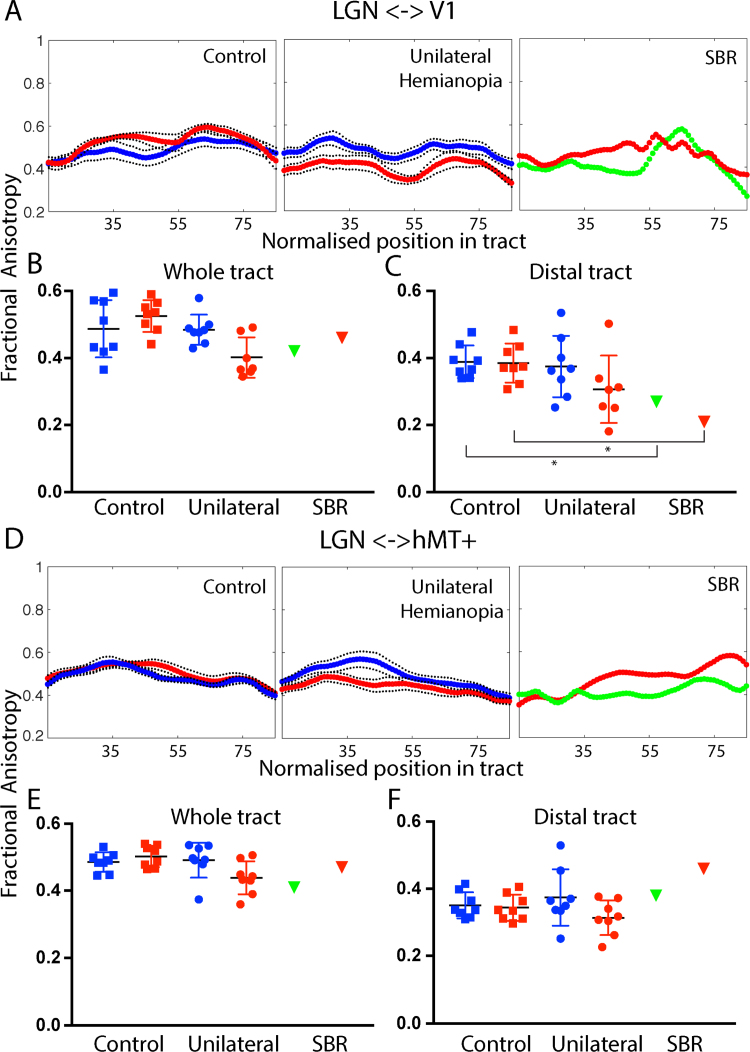
The upper section (panels A–C) shows the fractional anisotropy (FA) in the tract between LGN and V1. A shows the FA along the length of the tract from LGN to V1 for control participants (red: left hemisphere, blue: right hemisphere), patients with unilateral V1 damage (red: lesion hemisphere, blue: intact hemisphere) and SBR (red: left hemisphere, green: right hemisphere). B is the average FA across the whole tract for each group and hemisphere. C shows the average FA in the distal portion of the tract (nodes 85–100). The asterisk indicates that SBR's FA values are ≥ 2 sd. below the mean control value. (For interpretation of the references to color in this figure legend, the reader is referred to the web version of this article.)

The number of streamlines that can be tracked in a pathway can depend on multiple factors including the specific geometry of the cortex. Quantifying the microstructure of the pathways is therefore important to determine whether there is degeneration in the white matter. Fig. 5 shows the fractional anisotropy (FA) of tracts between LGN and V1 and LGN and hMT+. A shows the mean FA along the central 70% tract between LGN and V1 where 0 corresponds to the LGN and 100 to area V1 (nodes 15–85 are shown). In the control participants, red corresponds to the left hemisphere and blue to the right. There is little difference between FA in the two hemispheres. The patients with hemianopia show lower FA in the lesioned (red) compared to the intact (blue) hemisphere along the length of the pathway. Note that there are only 7 data points for the unilateral group in the lesioned hemisphere because one patient with a relatively large lesion did not show any streamlines between LGN and V1. SBR shows similar FA in both hemispheres with lower FA, particularly in the distal region close to V1.

The FA measured across the length of the tract is variable in all participant groups (B), but is comparable in SBR and the lesioned hemisphere of the patients with unilateral damage. Unsurprisingly, the FA drops off in the more distal portion of the tract (C), closer to the lesioned area. Indeed in SBR the FA in this region is ≥ 2 sd. below the mean control value.

Since the LGN <-> V1 tract and the LGN <-> hMT+ tract emerge from similar locations, much of the tract is common to both pathways at the resolution of the imaging. Therefore, it might be predicted that even if the specific fibres projecting to hMT+ are intact, the resolution of the tractography would mean that changes in the V1 tract would affect the microstructural measures. Thus, it is important to also consider the distal regions of the tract, where there is more divergence, as we have previously shown (Ajina et al., 2015b). The microstructure of the tract between LGN and hMT+ is shown in the lower section of Fig. 5 (panels D-F). Values for the FA in the two hemispheres are similar in control participants along the length of the path. As shown previously, in patients with unilateral hemianopia who show blindsight there is a difference between the two hemispheres in the proximal part of the tract, but much less so distally. SBR shows relatively high FA in the distal portion of the tract, while the more proximal regions are lower.

Thus, comparing the two tracts, LGN <-> V1 and LGN <-> hMT+, in SBR particularly, the largest discrepancy is in the distal sections, with a decrease in FA in the LGN <-> V1 tract near to V1 and an increase in the LGN <-> hMT+ tract near to hMT+.

The lower section of the figure (panels D-F) show the FA in the tract between LGN and hMT+ across the length of the tract (D) and averaged across the whole (E) and distal portion (F) of the tract. Error bars show standard deviations.

### 3.4 The transcallosal pathway between hMT+ in the two hemispheres is comparable to control participants

**Fig. 6:**
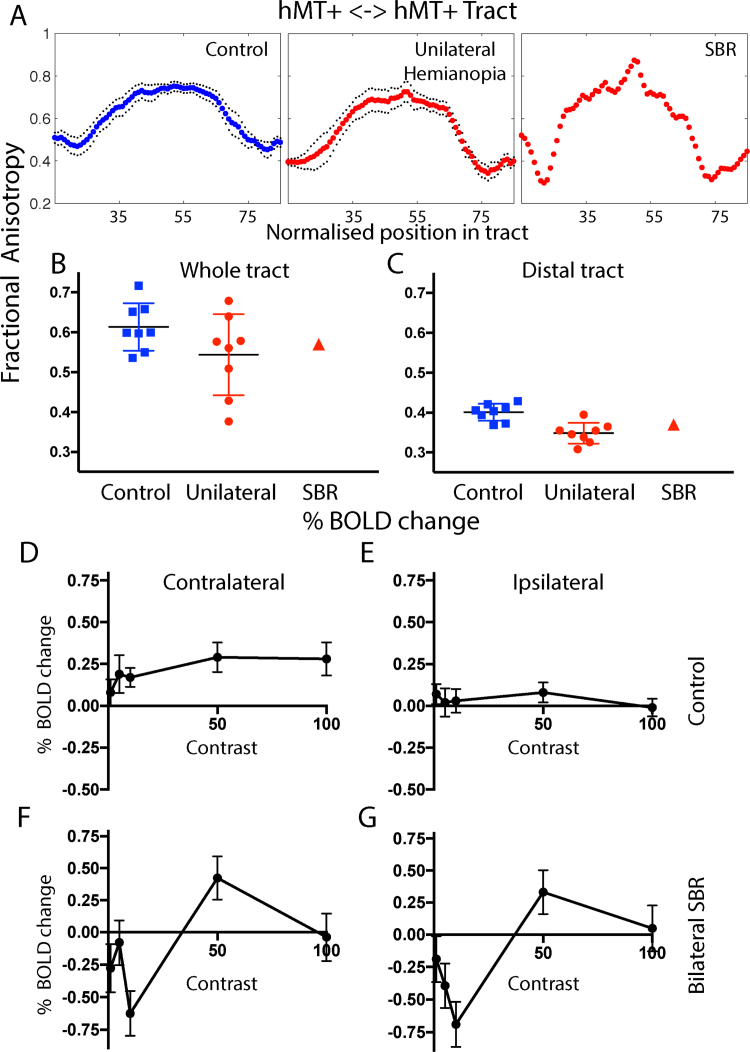
Communication between the two hemispheres may be important in residual vision. The microstructure of the connection between hMT+ in the two hemispheres is intact in SBR, with relatively high FA (A-C). In control participants there is a clear pattern of response to contrast in the contralateral hMT+ that saturates at low values. The ipsilateral response is considerably lower and shows a less clear pattern. Interestingly, in SBR the contralateral and ipsilateral responses to visual stimulation are very similar. Error bars show standard deviations.

Our previous work investigating the pattern of fMRI activation in patients with unilateral hemianopia has indicated that even hMT+ in the intact hemisphere has a slightly abnormal pattern of activity compared to the healthy cortex (Ajina et al., 2015a, Ajina et al., 2015c). Thus, it may be that increased reliance on hMT+ for residual vision alters connectivity between the two hemispheres. To quantify the microstructure of the tract between hMT+ in the two hemispheres, FA along the tract was measured, as shown in Fig. 6A–C. The FA values across control participants, patients with unilateral damage and SBR are similar, indicating that any damage does not appear to impinge on this pathway. In contrast to the LGN <-> hMT+ pathway, the distal regions tend to have lower FA in the patients, possibly due to the crossing of the damaged LGN <-> V1 pathway.

Ipsilateral responses can be generated during stimulus presentation in a single hemifield due to the large size of the receptive fields in hMT+ and communication across the corpus callosum. Fig. 6D shows the BOLD response to the different contrast levels in the contralateral hemisphere of healthy controls, which saturate at low values and are similar at 50% and 100% contrast. E shows the ipsilateral response, which is significantly lower and does not show any notable difference between contrast levels. In SBR, where responses are weaker (but also somewhat less reliable as they are from a single subject), the contralateral (F) and ipsilateral (G) responses are very similar, perhaps reflecting the lack of dominance of the contralateral hemisphere when V1 input is absent. This cannot be attributed to a global change in % BOLD, as a control ROI in the lateral ventricle showed a very different relationship with contrast (z = 2.9; p = 0.004).

## 4 Discussion

The residual vision and cortical changes evident in the case of bilateral cortical blindness presented here have much in common with those seen in patients with unilateral damage to V1. Behaviourally, both SBR and the patients with unilateral damage are able to detect stimuli of high contrast more successfully. MRI data demonstrate that the responses of hMT+ to high and low contrast stimuli and the tracts between LGN and hMT+ are comparable. The pathway connecting hMT+ in the two hemispheres also appears to be intact in all patients.

### 4.1 BOLD activity reflects perception in SBR

The previous study investigating hMT+ activation in SBR, measured the activation to a high contrast flickering chequerboard (Bridge et al., 2010). This study goes beyond that basic activity to determine the relationship between perceiving the stimulus and the BOLD response. Like the patients with unilateral damage to V1, the response was greater when the contrast was higher. This suggests that the BOLD signal change is related to the visibility of the stimulus, as with the healthy visual system.

### 4.2 Interhemispheric communication in the presence of bilateral V1 damage

SBR showed similar response patterns in the contra- and ipsi-lateral hemispheres, which was distinct from healthy controls. This suggests that inter-hemispheric communication between hMT+ was possible in spite of the bilateral V1 damage, supported by the presence of intact hMT+ connections using dMRI. Previous investigation of a patient with bilateral V1 damage found the corpus collosum had become significantly degenerated (de Gelder et al., 2008), with a severe loss of posterior callosal fibres. This was not the case here, however the functional significance of this intact pathway and any influence on visual perception remains unclear. In GY, a well-studied patient with unilateral V1 damage, transcranial magnetic stimulation could elicit a visual percept of phosphenes in the blind field, but only if stimulation was applied bilaterally over hMT+, and not if stimulation was restricted to ipsilesional hMT+ (Silvanto et al., 2007). Therefore, an interhemispheric connection may play a role in the experience of residual vision after V1 has been damaged.

### 4.3 SBR is younger than many of the unilateral patients and control participants

It is worth noting that SBR was a young man when he experienced his damage to V1, whereas the majority of stroke patients are considerably older. Both the BOLD response (D'Esposito et al., 1999) and FA (Inano et al., 2011) have been shown to decrease with normal aging. Thus, the relatively high FA seen in tracts in SBR may be related to his age in addition to preserved pathways. However, the comparison of tracts to hMT+ with the LGN <-> V1 tract suggests that in the presence of damage, the FA is still reduced below the level of the healthy older control participants.

### 4.4 Potential for rehabilitation in bilateral cortical blindness

Several lines of evidence suggest that this case of bilateral cortical blindness might be amenable to visual training to improve the residual vision. First, the patient is relatively young with white matter that has similar microstructure to healthy control participants. Thus, as with other types of training, there is potential for strengthening of white matter pathways (Scholz et al., 2009). Secondly, the damage to V1 is quite restricted, with no major involvement of the adjacent white matter. Thus, cortical areas outside of V1, in addition to hMT+ may also be available to increase visual function.

There are a number of approaches that could be adopted to improve function, including the motion training employed by the Huxlin group (Cavanaugh et al., 2017, Das et al., 2012, Das et al., 2014, Das and Huxlin, 2010, Martin et al., 2012) and training with Gabor patches used in the NeuroEye Therapy (Sahraie et al., 2013).

## 5 Conclusion

Patient SBR, with bilateral damage to V1, shows similar patterns of residual vision, hMT+ activation to visual stimulation and visual pathway microstructure as patients with unilateral damage. This suggests that the intact hemisphere is not required for residual vision, but rather visual information is conveyed via a direct pathway between LGN and hMT+.
